# Effects of aging on $T_{1}$, $T_{2}^{*}$, and QSM MRI values in the subcortex

**DOI:** 10.1007/s00429-016-1352-4

**Published:** 2017-02-06

**Authors:** M. C. Keuken, P.-L. Bazin, K. Backhouse, S. Beekhuizen, L. Himmer, A. Kandola, J. J. Lafeber, L. Prochazkova, A. Trutti, A. Schäfer, R. Turner, B. U. Forstmann

**Affiliations:** 1 Integrative Model-based Cognitive Neuroscience Research Unit, University of Amsterdam, Amsterdam, The Netherlands; 2 Netherlands Institute for Neuroscience, an Institute of the Royal Netherlands Academy of Arts and Sciences, Amsterdam, The Netherlands; 3 Max Planck Institute for Human Cognitive and Brain Sciences, Leipzig, Germany; 4 Siemens Healthcare GmbH, Diagnostic Imaging, Magnetic Resonance, Research and Development, Erlangen, Germany

**Keywords:** Ultra-high field 7 T MRI, $T_{1}$, $T_{2}^{*}$, QSM, Subcortex, Basal ganglia, Aging

## Abstract

The aging brain undergoes several anatomical changes that can be measured with Magnetic Resonance Imaging (MRI). Early studies using lower field strengths have assessed changes in tissue properties mainly qualitatively, using $T_{1}$- or $T_{2}^{*}$- weighted images to provide image contrast. With the development of higher field strengths (7 T and above) and more advanced MRI contrasts, quantitative measures can be acquired even of small subcortical structures. This study investigates volumetric, spatial, and quantitative MRI parameter changes associated with healthy aging in a range of subcortical nuclei, including the basal ganglia, red nucleus, and the periaqueductal grey. The results show that aging has a heterogenous effects across regions. Across the subcortical areas an increase of $T_{1}$ values is observed, most likely indicating a loss of myelin. Only for a number of areas, a decrease of $T_{2}^{*}$ and increase of QSM is found, indicating an increase of iron. Aging also results in a location shift for a number of structures indicating the need for visualization of the anatomy of individual brains.

## Introduction

Healthy aging across the adult lifespan is known to have a diverse effect on the anatomy of the brain. Historically, anatomical studies were performed on post-mortem specimens but with the development of magnetic resonance imaging (MRI) in vivo individual anatomy can now be visualized non-invasively. Several age-related findings which are frequently reported are the ventricular enlargement (e.g., Good et al. 2001; Raz and Rodrigue 2006; Greenberg et al. 2008; Keuken et al. 2013), changes in white matter microstructure (e.g., Walhovd et al. 2005; Benedetti et al. 2006; Raz and Rodrigue 2006), reduced grey matter volume (e.g., Courchesne et al. 2000; Good et al. 2001; Cherubini et al. 2009; Lemaitre et al. 2012), shifted location of gray matter nuclei (e.g., Dunnen and Staal 2005; Kitajima et al. 2008; Keuken et al. 2013; Mavridis et al. 2014), and an increase in iron deposition (e.g., Hallgren and Sourander 1958; Schenker et al. 1993; Zecca et al. 2004; Raz and Rodrigue 2006; Aquino et al. 2009; Pfefferbaum et al. 2009). These aging effects can vary locally across the brain (e.g., Greenberg et al. 2008; Cherubini et al. 2009; Draganski et al. 2011; Lemaitre et al. 2012). In addition to these anatomical changes, previous work shows changes in MRI parameters with age (e.g., Schenker et al. 1993; Steen et al. 1995; Courchesne et al. 2000; Good et al. 2001; Benedetti et al. 2006; Bastin et al. 2009; Saito et al. 2009; Cherubini et al. 2009; Draganski et al. 2011; Bilgic et al. 2012; Lebel et al. 2012; Lemaitre et al. 2012; Li et al. 2013; Yeatman et al. 2014; Lorio et al. 2014; Persson et al. 2015; Acosta-Cabronero et al. 2016; Betts et al. 2016). Changes in $T_{1}$ values are frequently reported and have been interpreted as changes in myelin structure (e.g., Silver et al. 1997; Maniega et al. 2015). Another common finding is the shortening of $T_{2}^{*}$ values, which have been interpreted as an indication of iron accumulation (Daugherty and Raz 2013).

The $T_{1}$ value indicates the recovery time of the longitudinal component of the magnetization following the application of a radio frequency excitation pulse. The quantitative $T_{1}$ values correlate highly with myelin content (Koenig 1991; Stüber et al. 2014) and can be used as an in vivo proxy for the underlying myeloarchitecture (Lutti et al. 2014; Dinse et al. 2015). The $T_{2}^{*}$ value indicates the decay of the transverse magnetization component as a result of proton interactions and magnetic field inhomogeneity. The $T_{2}^{*}$ values have been used as a proxy for iron, but recently several studies have demonstrated that the $T_{2}^{*}$ values are also strongly influenced by the presence and orientation of myelin (Fukunaga et al. 2010; Lee et al. 2010; Cohen-Adad et al. 2012; Stüber et al. 2014). Iron content in gray matter can be estimated more precisely using a post-processing technique called Quantitative Susceptibility Mapping (QSM) on the phase signal present in the $T_{2}^{*}$-weighted volumes (Schweser et al. 2011, 2016; Bilgic et al. 2012; Langkammer et al. 2012; Stüber et al. 2014; Ropele and Langkammer 2016). QSM quantifies the susceptibility distribution by estimating the magnetic field distribution, removing the background field contribution, and solves the inverse problem from field perturbation to magnetic susceptibility (Schweser et al. 2016).

Many age-related MRI studies have been limited by the use of low field strength or qualitative MRI sequences. Using low field strength such as 1.5 or 3T makes it difficult to visualize smaller nuclei such as the subthalamic nucleus or to discriminate the internal and external segment of the globus pallidus (e.g., Cho et al. 2008, 2010; Abosch et al. 2010; Beisteiner et al. 2011; Lenglet et al. 2012; Keuken et al. 2014).

The current study set out to describe the effects of aging on the volumetric and spatial properties, and to provide quantitative $T_{1}$, $T_{2}^{*}$, and QSM values at 7T static magnetic field for the striatum (STR), external segment of the globus pallidus (GPe), the internal segment of the globus pallidus (GPi), the red nucleus (RN), the subthalamic nucleus (STN), the substantia nigra (SN), and the periaqueductal grey (PAG). This was done by manually parcellating the different regions in structural ultra-high resolution 7T MRI data in three different age groups.

## Methods

### Participants

Thirty young participants with an average age of 23.8 years (age range 19–29; SD = 2.3, 14 females) which have been previously reported (Keuken et al. 2014) were included. In addition to the young participants, a second group of 14 middle-aged participants were scanned, with an average age of 52.5 years (age range 40–60, SD = 6.6, 7 females). Finally, a third group of 10 elderly participants were included, with an average age of 69.6 years (age range 60–75, SD = 4.6, 3 females). One elderly subject (male, age 73) did not complete all scans due to time constraints and was excluded from all further analyses. All subjects were right-handed, as confirmed by the Edinburgh Inventory (Oldfield 1971). None of the participants had a history of neurological disorders or currently suffered from psychiatric disorders as indicated by self-report and structured clinical interview. The study was approved by the local ethical committee of the Max Planck Institute for Human Brian and Cognitive Sciences in Leipzig, Germany.

### MRI acquisition

The structural data were acquired using a 7T Siemens Magnetom MRI scanner using a 24-channel head array Nova coil (NOVA Medical Inc., Wilmington MA) and consisted of three sequences: a whole brain MP2RAGE (Marques et al. 2010), a MP2RAGE covering a smaller slab, and a multi-echo 3D FLASH (Haase et al. 1986). The whole brain MP2RAGE had 240 sagittal slices with an acquisition time of 10:57 min (repetition time (TR) = 5000 ms; echo time (TE) = 2.45 ms; inversion times TI1/TI2 = 900/2750 ms; flip angle = 5°/3°; bandwidth = 250 Hz/Px; voxel size = 0.7 mm isotropic). The MP2RAGE slab consisted of 128 slices with an acquisition time of 9:07 min (TR = 5,000 ms; TE = 3.71 ms; TI1/TI2 = 900/2,750 ms; flip angle = 5°/3°; bandwidth = 240 Hz/Px; voxel size = 0.6 mm isotropic). The MP2RAGE sequence is a $T_{1}$-weighted structural scan but with the additional feature that it also provides a so-called $T_{1}$ map (Marques et al. 2010). The sequence is based on two volumes with different inversion times (the INV1 and INV2 volumes), which can be combined into a single $T_{1}$-weighted volume (UNI) or used to estimate the $T_{1}$ values. The resulting $T_{1}$ map gives reasonable estimates of the underlying $T_{1}$ values and has been shown to be highly reliable within subjects across scan sessions and scanners (Okubo et al. 2015; Voelker et al. 2016). It should, however, be noted that these $T_{1}$ maps may still contain residual transmit field bias (Marques and Gruetter 2013; Lutti et al. 2014).

The FLASH slab consisted of 128 slices with an acquisition time of 17:18 min (TR = 41 ms and three different TE: 11.22/20.39/29.57 ms; flip angle = 14°; bandwidth = 160 Hz/Px; voxel size = 0.5 mm isotropic). Both slab sequences consisted of axial slices tilted −23° to the true axial plane in scanner coordinates. See (Forstmann et al. 2014) for more information regarding the exact MRI parameters and data quality. All structural data have been made freely available and can be found on http://www.nitrc.org/projects/atag_mri_scans/ and http://datadryad.org/resource/doi:10.5061/dryad.fb41s.

### Manual parcellation of subcortical structures

The STR, GPe, GPi, RN, STN, and SN masks for the young group have been previously presented in (Keuken et al. 2014). For the young group, the PAG, lateral ventricle, third ventricle, cerebral aqueduct, and fourth ventricle were additionally parcellated and have not been published previously in the young group for this study. For the middle-aged and elderly group the STR, GPe, GPi, RN, STN, SN, PAG, lateral ventricle, third ventricle, cerebral aqueduct, and fourth ventricle were parcellated using the same parcellation protocol (Keuken et al. 2014). In short, the manual parcellation was performed using the FSL 4.1.4 viewer (http://fsl.fmrib.ox.ac.uk/fsl/fslview/) by two independent raters. Based on previous results (Keuken et al. 2014) the STR was segmented on the UNI MP2RAGE slab volumes, the STN, SN, and RN on the FLASH volumes, and finally the GPe and GPi on the QSM volumes. The PAG and ventricle system were parcellated on the UNI MP2RAGE whole brain volumes. The resolution and lack of contrast of the in vivo MP2RAGE scans made it very difficult to distinguish the different sub compartments of the STR (Voorn et al. 2004; Haber and Gdowski 2004; Neto et al. 2008; Haber and Knutson 2009; Keuken et al. 2014). Therefore, the STR was segmented as a whole without attempting to make distinctions between the different subparts which would have to be based on surrounding landmarks. The GPe and GPi were segmented on the QSM images as the $T_{2}^{*}$ image quality was not sufficient to clearly separate the interlamina between the GPe and GPi, whereas this was the case for the QSM (Keuken et al. 2014).

After parcellation of the structure, the inter-rater agreement was assessed using the Dice coefficient (Dice 1945). The resulting conjunction masks were used for further analysis unless stated otherwise. All volume estimates are calculated in the native space of the MRI sequence in which the structure was parcellated. The intracranial volume estimate was calculated using the skull stripped whole brain MP2RAGE unified volume, using BET (Smith 2002). Because the whole brain MP2RAGE scan was skull stripped automatically, no inter-rater agreement was assessed.

Due to the time-consuming nature of manual parcellation, it was not possible to have the same raters for the middle-aged and elderly group as in the young group that was published previously (Keuken et al. 2014). Therefore, it might be the case that some of the effects of aging are actually due to different raters for the young age group versus the middle-aged and elderly group. To test whether this could influence the general conclusion, the same raters that segmented the RN and STN for the middle-aged and elderly group, redid the parcellation for the young RN and STN masks, which allowed testing the influence on the results of different raters for different age groups.

### Registration to standard stereotactic MNI-space

**Fig. 1:**
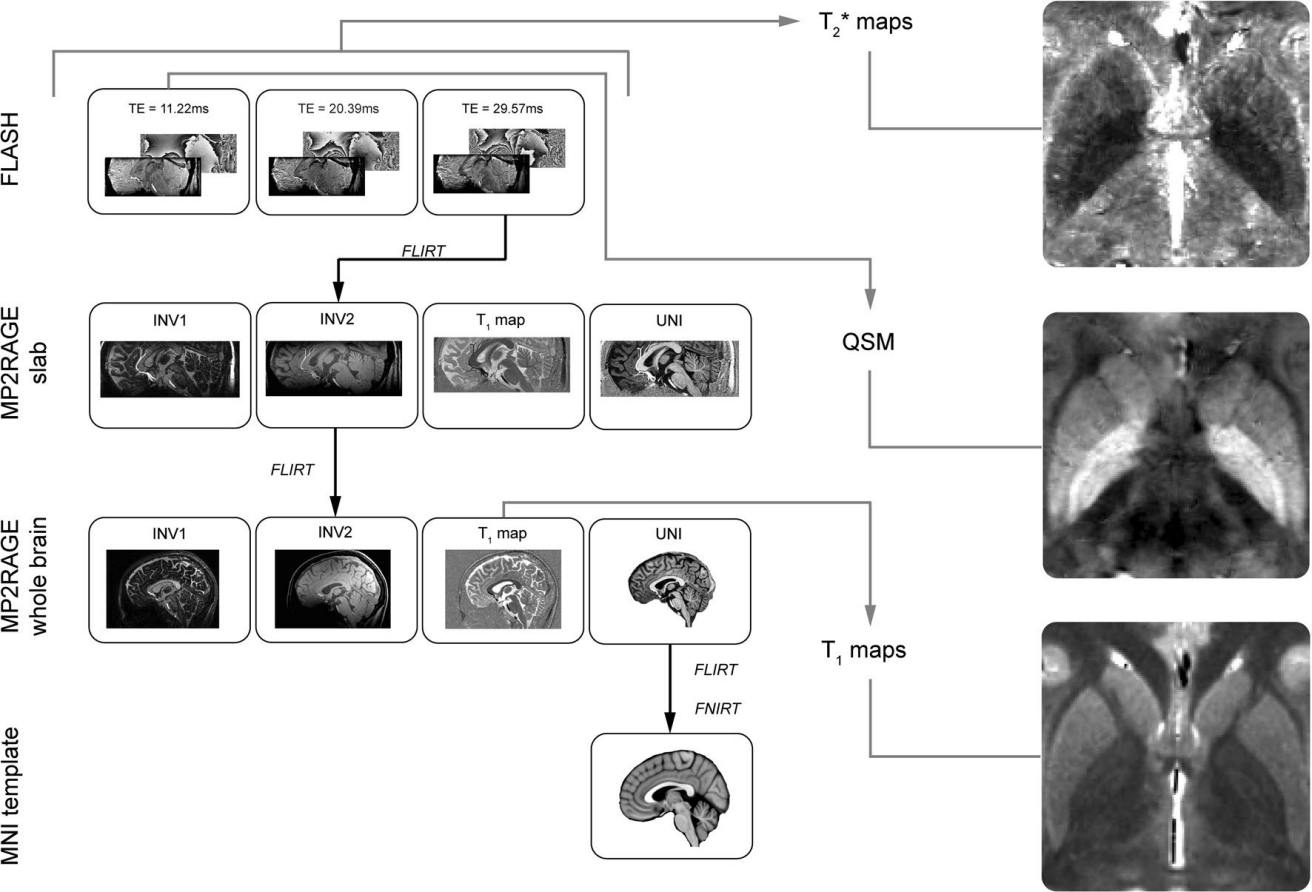
The registration pipeline. The RN, STN, and SN were parcellated on the FLASH volumes. The GPe and GPi were parcellated on the QSM volumes which were based on the first echo time of the FLASH volumes. The STR was parcellated on the MP2RAGE slab UNI volume whereas the ventricle compartments and the PAG were parcellated on the MP2RAGE whole brain UNI volume. Using the resulting transformation matrices, the conjunction masks were transformed to the $T_{2}^{*}$, QSM, or $T_{1}$ map space so that the quantitative MRI values could be extracted. Examples of the $T_{2}^{*}$, QSM, and $T_{1}$ maps of a young participant are shown on the* right*

The third echo time of the FLASH sequence was linearly registered to the MP2RAGE slab second inversion volume using Mutual information and 6 Degrees of Freedom (DoF). The MP2RAGE slab second inversion volume was registered to the MP2RAGE whole brain second inversion volume using correlation ratio information and 6 DoF. The MP2RAGE whole brain unified volumes were registered to the MNI152 0.5 mm template using correlation ratio information and 12 DoF. All linear registrations used tri-linear interpolation and were done using FLIRT (Jenkinson and Smith 2001; Jenkinson et al. 2002) as implemented in FSL 5.0.2. Prior to the registration to MNI standard space, both the MP2RAGE whole brain and MNI152 template were skull stripped using BET (Smith 2002). Finally, the MP2RAGE whole brain unified volumes were non-linearly registered to the MNI152 0.5 mm template using the linearly registered volumes as input for FNIRT in combination with the default settings of FNIRT (Andersson et al. 2007; Jenkinson et al. 2012). The FLASH and MP2RAGE slab images were transformed to the MNI152 template by combining the resulting transformation matrices from the FLASH to MP2RAGE slab, MP2RAGE slab to MP2RAGE whole brain, and MP2RAGE whole brain to MNI152 registration. All individual registration steps were visually checked for misalignment. All parcellated structures were transformed to the MNI152 template using the corresponding transformation matrices and added together to create a probabilistic atlas of each structure separately. This was done both for the linear and non-linearly registered structures. All the probabilistic atlases are made freely available and can be found on https://www.nitric.org/projects/atag/. See Fig. 1 for a flowchart of the registration pipeline.

**Fig. 2:**
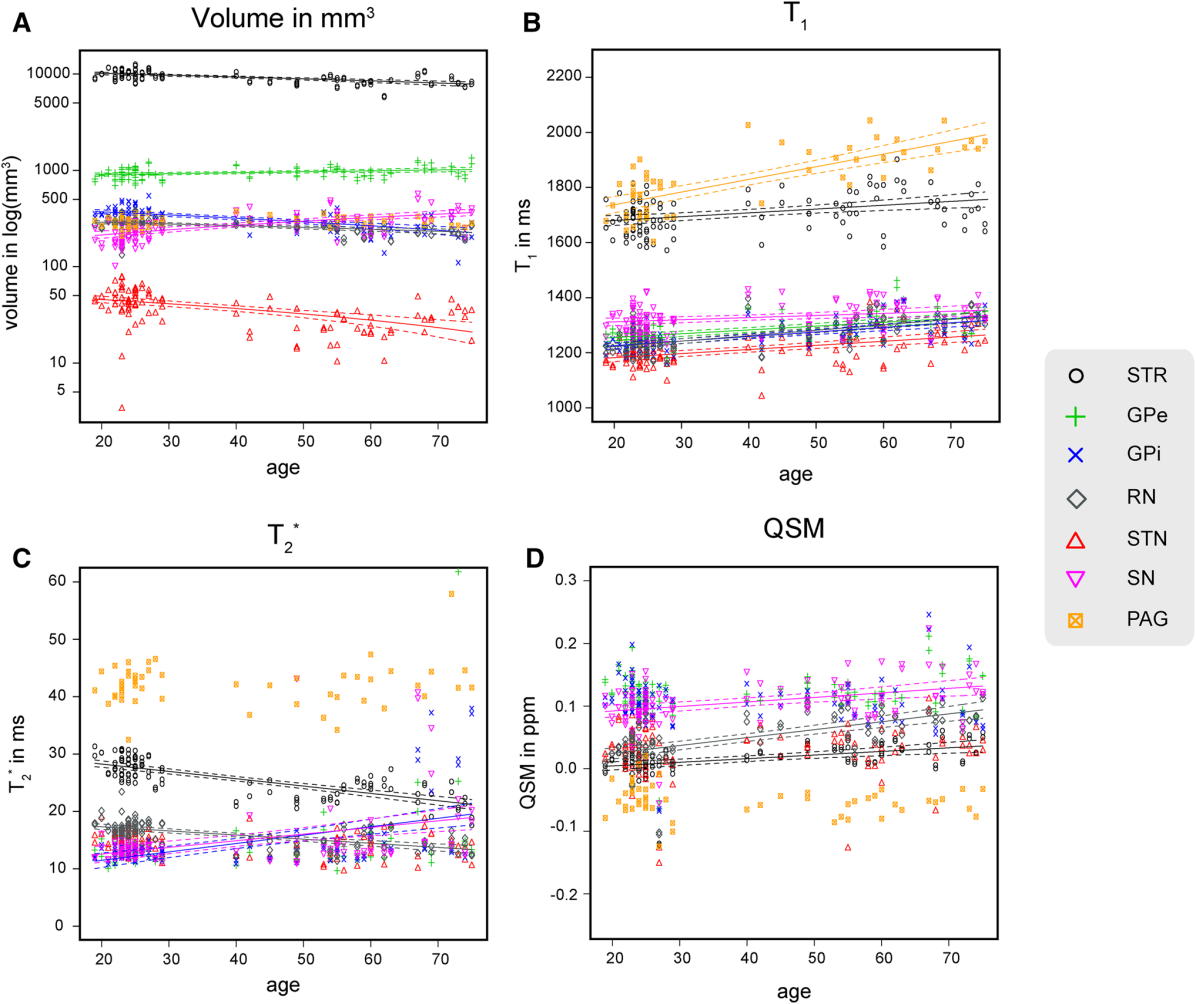
The volume, $T_{1}$, $T_{2}^{*}$, and QSM values per structure for each individual participant. The regression lines indicate a significant correlation with age. The* dotted lines* indicate the 95% confidence interval. *STR* striatum, *GPe* globus pallidus externa, *GPi* globus pallidus interna, *RN* red nucleus, *STN* subthalamic nucleus, *SN* substantia nigra, *PAG* periaqueductal* grey*

**Fig. 3:**
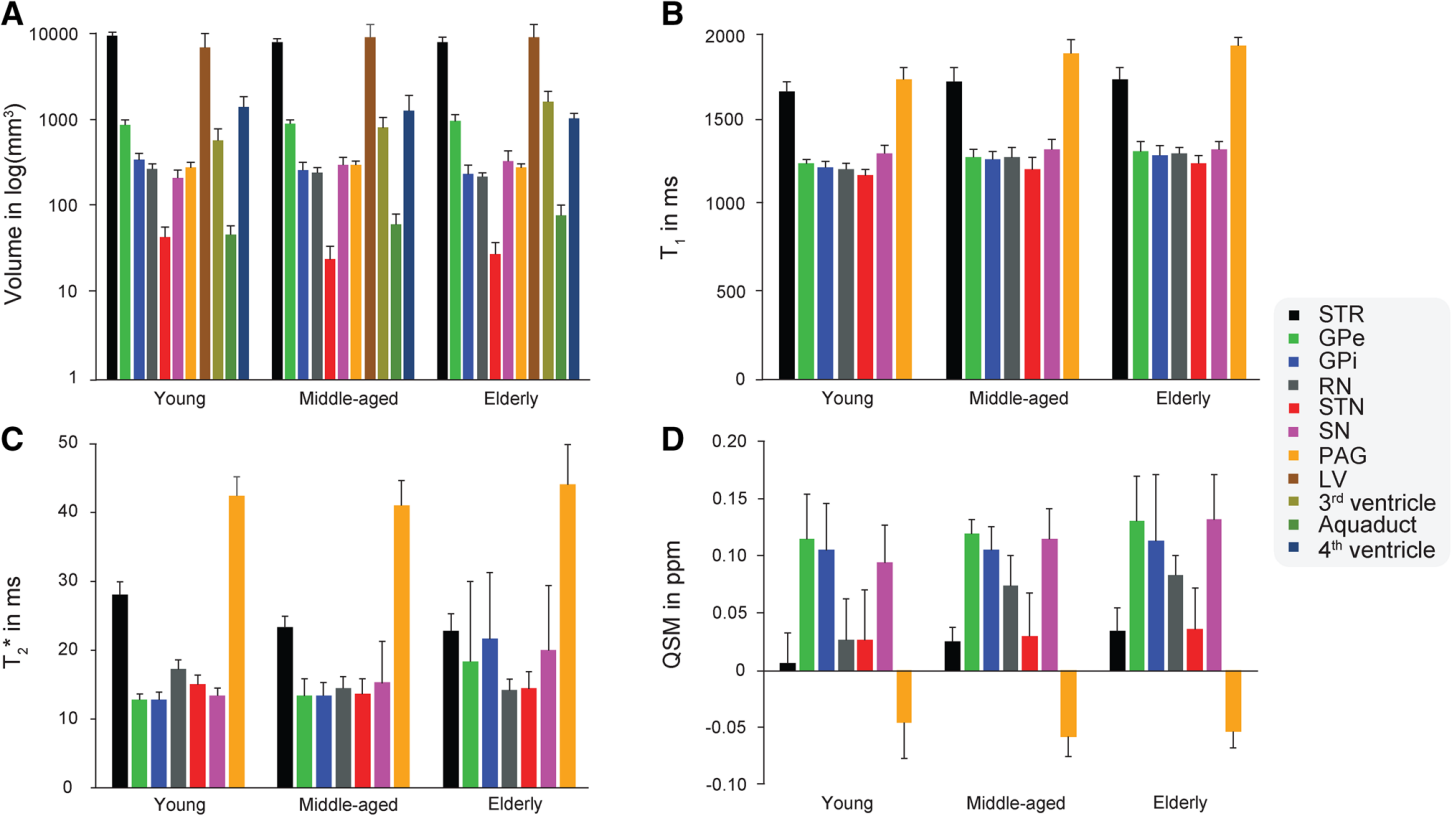
The average volume, $T_{1}$, $T_{2}^{*}$, and QSM values across hemispheres per structure for each age group. The error bars indicate 1 sd. *STR* striatum, *GPe* globus pallidus externa, *GPi* globus pallidus interna, *RN* red nucleus, *STN* subthalamic nucleus, *SN* substantia nigra, *PAG* periaqueductal grey, *LV* lateral ventricle

Statistical analyses were done in R 3.2.4 (http://www.r-project.org; R Development Core Team 2013). All correlation and partial correlation tests were done using a two-sided Pearson’s *r* test with a critical α of 0.05 using the individual age as a continuous variable. The three age groups were only used as a discrete variable to provide the summary statistics. A Bonferroni correction was used to correct for multiple comparisons per family of tests. For instance, the Bonferroni correction for the correlations between age and the Dice coefficient was based on 11 tests which correspond to the number of structures for which the Dice coefficient was calculated. All reported *p* values are after Bonferroni correction. For all significant correlations, 95% confidence intervals were estimated using the function *predict* as implemented in R and plotted in Figs. 2 and 3

### Effect of age on location

To test whether age had an effect on the location of the gray matter nuclei in standard MNI space, the following analysis was done. First, the Center of Mass (CoM) was calculated for each linear registered structure using FSLUTILS (FSL 5.0.2). To reduce the number of tests, a Principal Component Analysis (PCA) was computed on the X, Y, and Z CoM coordinates of each individual structure using *princomp* in R Team (2013). Since we had no a-priori hypothesis regarding lateralization and age, the negative X-coordinate values, corresponding to the left hemisphere, were converted to positive values and combined in a single PCA analysis. The resulting first principal component corresponds to a new latent variable which captures the maximal amount of variance. Finally, the eigenvector scores of the first principal component of each structure and participant were correlated with age. This analysis allows us to test whether there is a relationship between the individual spatial location, as indicated by the individual eigenvector scores, and age.

### Computation of the $T_{1}$, $T_{2}^{*}$, and QSM values

The $T_{1}$ values for the STR, GPe, GPi, RN, STN, SN, and PAG were extracted from the MP2RAGE whole brain $T_{1}$ map. The $T_{2}^{*}$ values for the STR, GPe, GPi, RN, STN, SN, and PAG were calculated using the $T_{2}^{*}$ fitting module as implemented in the CBS High-Res Brain Processing Tools for MIPAV (http://www.nitrc.org/projects/cbs-tools/) (Bazin et al. 2013). This module uses a nonlinear least squares function to estimate a single-component $T_{2}^{*}$ map (Whittall et al. 1997):

$$S(\text{TE})={{S}_{0}}{{e}^{(-\text{TE}/T_{2}^{*})}}.$$

Possibly due to the interpolation between different MRI scans or intrinsic partial voluming, the STR and PAG masks contained voxels which, based on the $T_{1}$, $T_{2}^{*}$ values, and the spatial location, belonged to the lateral ventricle or cerebral aqueduct. Extracting the mean $T_{1}$ and $T_{2}^{*}$ values from these voxels would result in a skewed estimate given that the $T_{1}$ and $T_{2}^{*}$ values of the CSF are considerably distinct from those in the gray matter ROIs (McRobbie et al. 2006). We addressed this by calculating the mean $T_{1}$ and $T_{2}^{*}$ values of the ventricle compartment or the cerebral aqueduct and used that value as an upper threshold when extracting the mean $T_{1}$ and $T_{2}^{*}$ values for the STR and PAG. For completeness we also tested the effects of aging on the non-thresholded $T_{1}$ and $T_{2}^{*}$ values for the STR and PAG.

The QSM was calculated using the phase information of the first echo time of the FLASH acquisition and the method proposed by Schweser et al. (2012). The first echo time was chosen because it had the highest absolute SNR. The coil combination of phase data was done automatically by the scanner vendor software (version VB17). This automatic coil combination results in some minor phase singularities, but are partially accounted by the employed superfast dipole inversion (SDI) approach which, includes a modified SHARP algorithm. The modified SHARP algorithm is described in Schweser et al. (2012). The masking of the data was done using BET which was manually adjusted if the binary mask was too lenient. This was determined by visual inspection on a subject to subject basis. The QSM intensities were normalized by subtracting the mean QSM value of the combined lateral ventricle conjunction masks from the main QSM volume. This normalisation was necessary due to the arbitrary settings of the resonance frequency, use of high pass filtering, and the use of a single echo to calculate the QSM values (Schäfer et al. 2009; Langkammer et al. 2012). To ensure that no gray matter tissue was included in the lateral ventricle masks, the masks were first eroded with a 2 mm Gaussian kernel. All $T_{1}$-, $T_{2}^{*}$-maps, and QSM were calculated in native space.

## Results

### Inter-rater reliability

All segmentations resulted in a good to excellent mean agreement between the two raters, indicating that it is feasible to identify these subcortical structures in individual space. See Table 1 for the Dice coefficient per structure and age group. There were, however, different effects of age on the inter-rater reliability for these separate structures. For the STR, RN, SN, LV, and third ventricle there were positive correlations between the Dice coefficient and age (STR: *r* = 0.58, t(104) = 7.22, *p* < 0.001; RN: *r* = 0.48, t(104) = 5.57, *p* ≤ 0.001; SN: *r* = 0.33, t(104) = 3.59, *p* = 0.006; third ventricle: *r* = 0.63, t(51) = 5.75, *p* < 0.001), indicating that the inter-rater reliability increases with age. This was the opposite for the GPe and the GPi as there was a negative correlation between the Dice coefficient and age (GPe: *r* = −0.46, t(104) = −5.30, *p* < 0.001; GPi: *r* = −0.54, t(104) = −6.62, *p* < 0.001). There was no significant correlation between the Dice coefficient and age for the STN, PAG, LV, cerebral aqueduct, and fourth ventricle.

**Table 1:** The inter-rater reliability coefficient and conjunction volume in mm^3^ estimates

Structure	Young				Middle aged				Elderly			
	Volume		Dice coefficient		Volume		Dice coefficient		Volume		Dice coefficient	
	Mean mm^3^	SD	Mean	SD	Mean mm^3^	SD	Mean	SD	Mean mm^3^	SD	Mean	SD
Intracranial volume	1545970.0	113255.60	–	–	1523494.0	117079.20	–	–	1546127.0	17295930.0	–	–
STR												
Left	10128.62‡	1038.96	0.89	0.01	8586.88	903.45	0.9	0.01	8466.28	1334.86	0.91	0.01
Right	10064.2‡	1051.34	0.89	0.01	8423.03	723.4	0.89	0.01	8370.9	1506.64	0.91	0.01
Overall	10096.41‡	1036.78	0.89	0.01	8504.95	807.42	0.9	0.01	8418.59	1381.72	0.91	0.01
GPe												
Left	932.47‡	124.16	0.87	0.02	972.34	115.55	0.83	0.08	1056.35	181.07	0.85	0.02
Right	904.5‡	123.09	0.88	0.01	963.31	108.36	0.86	0.03	1007.08	143.92	0.84	0.03
Overall	918.49‡	123.38	0.88	0.02	967.84	110.02	0.84	0.06	1033.59	160.87	0.84	0.02
GPi												
Left	366.63‡	63.16	0.82	0.03	267.98	53.31	0.77	0.07	244.82	47.06	0.74	0.08
Right	365.11‡	57.42	0.84	0.03	286.31	63.26	0.8	0.05	248.19	90.29	0.75	0.08
Overall	365.87‡	59.85	0.83	0.03	277.15	58.16	0.79	0.06	247.01	69.85	0.74	0.08
RN_1												
Left	285.05‡	49.42	0.89	0.03	–	–	–	–	–	–	–	–
Right	276.85‡	49.79	0.89	0.03	–	–	–	–	–	–	–	–
Overall	280.95	49.36	0.89	0.03	–	–	–	–	–	–	–	–
RN_2												
Left	290.76	40.39	0.88	0.03	260.69	35.21	0.92	0.02	239.07	33.38	0.91	0.02
Right	279.1	43.58	0.89	0.02	253.35	38.02	0.93	0.02	228.93	31.84	0.92	0.01
Overall	284.93	42.07	0.89	0.03	257.02	36.15	0.92	0.02	234.0	32.07	0.92	0.02
STN_1												
Left	52.83‡	16.26	0.72	0.14	–	–	–	–	–	–	–	–
Right	59.5‡	15.71	0.76	0.09	–	–	–	–	–	–	–	–
Overall	56.17‡	16.2	0.74	0.12	–	–	–	–	–	–	–	–
STN_2												
Left	40.75	12.05	0.66	0.14	25.46	9.07	0.72	0.07	29.71	8.95	0.71	0.09
Right	50.07	14.53	0.71	0.14	24.99	9.19	0.7	0.09	28.72	11.46	0.68	0.1
Overall	45.41	14.04	0.68	0.14	25.22	8.96	0.71	0.08	29.22	9.99	0.69	0.09
SN												
Left	223.18‡	46.79	0.76	0.05	319.55	73.21	0.79	0.04	369.72	114.84	0.79	0.07
Right	226.33‡	50.46	0.76	0.04	313.26	77.96	0.79	0.07	331.83	97.77	0.79	0.04
Overall	224.75‡	48.27	0.76	0.04	316.4	74.28	0.79	0.06	350.78	105.28	0.79	0.06
PAG	292.25	40.47	0.79	0.04	315.76	33.76	0.82	0.03	292.05	28.59	0.78	0.04
LV												
Left	7829.75	3367.64	0.89	0.03	10001.19	3844.15	0.89	0.02	13728.48	3657.5	0.91	0.02
Right	7220.83	3579.82	0.87	0.04	9579.08	3776.74	0.87	0.05	14322.18	5622.29	0.91	0.02
Overall	7525.29	3459.43	0.88	0.04	9790.54	3745.54	0.88	0.04	14025.33	4611.28	0.91	0.02
3rd	606.47	205.54	0.69	0.07	859.32	263.74	0.76	0.06	1756.09	536.94	0.82	0.03
Aquaduct	49.52	12.43	0.72	0.06	63.72	20.95	0.73	0.05	82.55	25.47	0.76	0.05
4th	1514.64	521.67	0.74	0.11	1356.6	641.71	0.75	0.07	1091.12	178.11	0.66	0.12

Rater 1 was constant across structures and participants

*STR* striatum, *GPe* globus pallidus externa, *GPi* globus pallidus interna, *RN* red nucleus, *STN* subthalamic nucleus, *SN* substantia nigra, *PAG* periaqueductal grey, *LV* lateral ventricle, *3rd* third ventricle, *Aqueduct* cerebral aqueduct, *4th* fourth ventricle. *_1* rater pair between rater 1 and rater 2; *_2* rater pair between rater 1 and rater 3

^‡^Previously published in Keuken et al. (2014)

The variability in inter-rater reliability between the structures and age ranges could be considered a confounding factor for any further analysis. We addressed this by incorporating the Dice coefficient as a covariate in all further analyses unless stated otherwise.

### Effect of different raters

The correlation between the Dice coefficient and age for the STN and RN did not statistically change whether the previously published masks were used or the new parcellation (RN: *r* = 0.45, t(104) = 5.18, *p* < 0.001 versus *r* = 0.48, t(104) = 5.57, *p* < 0.001; STN: *r* = −0.18, t(104) = −1.99, *p* = 0.062 versus  *r* = 0.07, t(104) = 0.75, *p* =  0.46 respectively). As indicated by the Fisher *r*-to-*z* transformation (Steiger 1980), the correlations did not statistically differ (RN: *Z* = −0.27, *p* = 0.79; STN: *Z* = −1.81, *p* = 0.07). The volumetric effects of age for the RN and the STN were also independent of which masks were used for the young group (RN: *r* = −0.55, t(106) = −6.73, *p* < 0.001 versus *r* = −0.53, t(106) = −6.26, *p* < 0.001; STN: *r* = −0.70, t(106) = −9.89, *p* < 0.001 versus *r* = −0.67, t(106) = −9.11, *p* < 0.001, respectively). As indicated by the Fisher r-to-z transformation, the correlations did not statistically differ (RN: *Z* = −0.2, *p* = 0.84; STN: *Z* = −0.41 *p* = 0.68).

These results indicate that the effect of rater pair is minimal compared to the effect of age since the Dice coefficient and volumetric results with age did not statistically change depending on the rater pair. All results that follow which include the RN and STN masks of the young participants are based on the rater pair who also parcellated the middle-aged and elderly participants.

### Effect of aging on volume

In line with previous work (Courchesne et al. 2000; Ge et al. 2002; Mortamet et al. 2005), there was no significant effect of age on the intracranial volume. The STR, GPi, RN, and STN all showed a decrease in volume with age (STR: *r* = −0.65, t(106) = −8.68, *p* < 0.001; GPi: *r* = −0.40, t(106) = −4.44, *p* < 0.001; RN: *r* = −0.53, t(106) = −6.26, *p* < 0.001 STN: *r* = −0.67, t(106) = −9.11, *p* < 0.001). However, somewhat unexpectedly, both the GPe and SN showed a positive correlation between volume and age (*r* = 0.33, t(106) = 3.535, *p* = 0.007; *r* = 0.53, t(106) = 6.33, *p* < 0.001). While positive relations between grey matter volume and age have been previously reported in the literature (Mueller et al. 1998; Salat et al. 2002, 2004; Lemaitre et al. 2012) these are usually interpreted as an artefact of the employed method or measurement error. While we have no reason to assume that the employed MRI sequence and segmentation protocol were biased towards the GPe and SN, the volumetric results are counterintuitive and should be interpreted with caution. In line with previous work (Fjell and Walhovd 2010), the LV, third ventricle and the cerebral aqueduct showed a significant increase in volume with age (LV: *r* = 0.54, t(106) = 6.51, *p* < 0.001; third ventricle: *r* = 0.45, t(53) = 3.56, *p* = 0.009; cerebral aqueduct: *r* = 0.52, t(53) = 4.36, *p* < 0.001), whereas the PAG and the fourth ventricle did not show a significant increase in volume with age. Overall, the total volume of the parcellated ventricle compartments indicated an increase with age (*r* = 0.47, t(53) = 3.81, *p* < 0.001). See Fig. 2a for the volumetric results of the gray matter nuclei; Table 1 and Fig. 3a for the average values per age group.

### Effect of aging on the location

To test whether aging had an effect on the spatial location of the different gray matter structures, the scores along the first eigenvector of the PCA analysis were correlated with the age of the participants while controlling for the Dice coefficient and the total ventricle volume. The total ventricle volume was included as a covariate since age and ventricle volume were strongly correlated, and ventricular expansion may affect the location of subcortical nuclei. In line with previous work there were several structures that showed a significant effect of age on the spatial location in standard MNI-space (STR: *r* = 0.29, t(106) = 3.05, *p* = 0.021; GPe: *r* = 0.47, t(106) = 5.35, *p* < 0.001; STN: *r* = 0.28, t(106) = 2.91, *p* = 0.031; RN: *r* = 0.28, t(106) = 2.90, *p* = 0.032; PAG: *r* = 0.28, t(106) = 2.94, *p* = 0.028) indicating the need for the visualization of the individual anatomy in aging populations (Keuken et al. 2013).

### Effects of aging on $T_{1}$ values

All grey matter nuclei showed a positive correlation between the $T_{1}$ values and age (STR: *r* = 0.32, t(106) = 3.41, *p* = 0.006; GPe: *r* = 0.44, t(106) = 4.97, *p* < 0.001; GPi: *r* = 0.47, t(106) = 5.43, *p* < 0.001; STN: *r* = 0.50, t(106) = 5.78, *p* < 0.001; RN: *r* = 0.60, t(106) = 7.57, *p* < 0.001; SN: *r* = 0.33, t(106) = 3.53, *p* = 0.004; PAG: *r* = 0.77, t(53) = 8.54, *p* < 0.001). The non-thresholded $T_{1}$ values of the STR and PAG also showed a positive correlation with age (STR: *r* = 0.26, t(106) = 2.73, *p* = 0.015; PAG: *r* = 0.77, t(53) = 8.54, *p* < 0.001). An increase in $T_{1}$ values is thought to reflect a decrease of myelinisation (Callaghan et al. 2014). See Fig. 2b for the $T_{1}$ values per structure; Table 2 and Fig. 3b for the average values per age group.

**Table 2:** The mean and standard deviation of the $T_{1}$ values in ms per structure and age group

Structure	Young		Middle-aged		Elderly	
	Mean	SD	Mean	SD	Mean	SD
STR						
Left	1643.71	39.53	1703.71	71.78	1721.40	62.53
Right	1723.51	41.30	1762.79	70.91	1774.38	73.01
Overall	1683.61	56.79	1733.25	76.20	1747.01	71.35
GPe						
Left	1245.14	28.61	1287.44	48.81	1328.26	64.87
Right	1277.72	30.54	1304.99	45.35	1344.61	63.03
Overall	1261.43	33.62	1296.22	47.09	1336.44	62.61
GPi						
Left	1223.43	32.29	1279.28	48.88	1310.55	56.11
Right	1241.45	31.76	1284.55	41.49	1316.10	55.39
Overall	1232.44	33.03	1281.91	44.57	1313.33	54.16
RN						
Left	1235.49	38.80	1296.25	61.35	1319.50	30.74
Right	1225.58	34.18	1291.36	55.81	1315.22	30.16
Overall	1230.54	36.60	1293.80	57.60	1317.36	29.62
STN						
Left	1182.41	36.77	1215.14	74.46	1238.13	49.13
Right	1202.12	39.10	1228.43	74.75	1292.45	39.81
Overall	1192.26	38.92	1221.79	73.52	1265.29	51.61
SN						
Left	1303.52	43.87	1338.69	55.87	1337.68	48.30
Right	1326.41	40.16	1353.00	68.91	1355.96	48.97
Overall	1314.97	43.27	1345.84	61.98	1346.82	48.12
PAG	1750.91	74.30	1907.00	84.29	1952.60	47.68

*STR* striatum, *GPe* globus pallidus externa, *GPi* globus pallidus interna, *RN* red nucleus, *STN* subthalamic nucleus, *SN* substantia nigra, *PAG* periaqueductal grey

### Effects of aging on $T_{2}^{*}$ values

The STR and RN showed a negative correlation between the $T_{2}^{*}$ values and age (STR: *r* = −0.71, t(106) = −10.15, *p* < 0.001; RN: *r* = −0.49, t(106) = −5.74, *p* < 0.001). Contrary to these structures, there was a positive correlation between the $T_{2}^{*}$ values and age for the GPi and SN, two of which were found to have an increased volume with age (GPi: *r* = 0.46, t(106) = 5.28, *p* < 0.001; SN: *r* = 0.34, t(106) = 3.65, *p* < 0.001). The STN, GPe, and PAG did not show a significant correlation between age and $T_{2}^{*}$ values. The $T_{2}^{*}$ values for the non-thresholded STR indicated a negative correlation with age, whereas the PAG still did not show a significant correlation (STR: *r* = −0.55, t(106) = −6.62, *p* < 0.001). A change in $T_{2}^{*}$ values is thought to reflect a change in ratio of the contribution of myelin or iron to the measured signal (Deistung et al. 2013; Stüber et al. 2014). See Fig. 2c for the $T_{2}^{*}$ values per structure; Table 3 and Fig. 3c for the average values per age group.

**Table 3:** The mean and standard deviation of the $T_{2}^{*}$ values in ms per structure and age group

Structure	Young		Middle-aged		Elderly	
	Mean	SD	Mean	SD	Mean	SD
STR						
Left	27.97	1.98	23.42	1.64	23.38	2.70
Right	28.00	1.89	23.23	1.63	22.02	2.52
Overall	27.97	1.92	23.33	1.61	22.70	2.63
GPe						
Left	12.95	1.20	13.56	2.62	20.07	15.89
Right	12.59	0.83	13.15	2.32	16.39	5.15
Overall	12.77	1.04	13.36	2.43	18.22	11.62
GPi						
Left	12.82	0.85	13.34	2.21	20.95	9.41
Right	12.65	0.92	13.07	1.57	22.26	10.86
Overall	12.74	0.88	13.20	1.88	21.61	9.88
RN						
Left	17.38	1.67	14.26	1.61	14.29	1.65
Right	17.14	1.30	14.69	1.83	14.00	1.90
Overall	17.26	1.49	14.48	1.70	14.15	1.73
STN						
Left	15.11	1.60	13.39	2.46	14.41	2.78
Right	14.98	1.45	13.61	2.11	14.41	2.85
Overall	15.05	1.51	13.50	2.25	14.41	2.73
SN						
Left	13.32	1.11	14.23	2.36	20.55	10.19
Right	13.12	0.94	16.00	8.20	19.37	9.22
Overall	13.22	1.03	15.12	5.99	19.96	9.44
PAG	42.31	2.84	40.85	3.50	43.92	5.73

*STR* striatum, *GPe* globus pallidus externa, *GPi* globus pallidus interna, *RN* red nucleus, *STN* subthalamic nucleus, *SN* substantia nigra, *PAG* periaqueductal grey

### Effects of aging on the QSM values

**Fig. 4:**
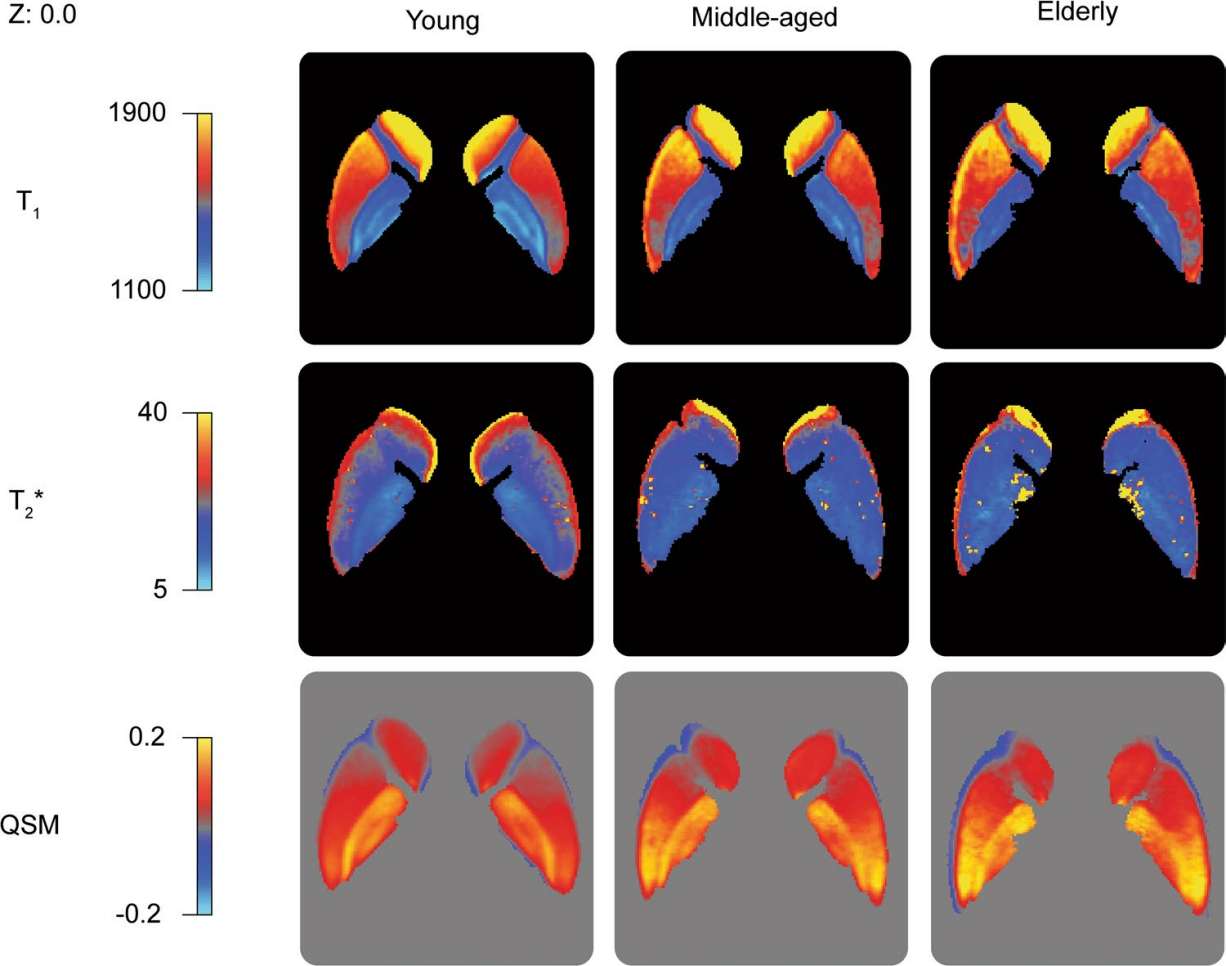
The spatial distribution of the mean $T_{1}$, $T_{2}^{*}$, and QSM values within the striatum and globus pallidum per age group at MNI Z coordinate 0. The linearly registered probability atlas was thresholded at 33% overlap

The STR, RN, and SN showed a positive correlation of age with the QSM values indicating an increase of iron concentration (STR: *r* = 0.43, t(106) = 4.80, *p* < 0.001; RN: *r* = 0.45, t(106) = 5.12, *p* < 0.001; SN: *r* = 0.37, t(106) = 3.974, *p* = 0.001). There was no statistically significant relationship between the QSM values and age for the GPe, GPi, STN or PAG. See Fig. 2d for the QSM values per structure; Table 4 and Fig. 3d for the average values per age group. See Fig. 4 for the spatial distribution of mean $T_{1}$, $T_{2}^{*}$, and QSM values within the striatum and globus pallidum per age group.

**Table 4:** The mean and standard deviation of the QSM concentration in ppm per structure and age group

Structure	Young		Middle-aged		Elderly	
	Mean	SD	Mean	SD	Mean	SD
STR						
Left	0.003	0.027	0.026	0.014	0.035	0.019
Right	0.010	0.029	0.025	0.011	0.035	0.019
Overall	0.007	0.028	0.025	0.012	0.035	0.019
GPe						
Left	0.111	0.038	0.120	0.015	0.130	0.044
Right	0.119	0.040	0.120	0.011	0.133	0.039
Overall	0.115	0.039	0.120	0.013	0.131	0.040
GPi						
Left	0.100	0.038	0.100	0.020	0.110	0.066
Right	0.111	0.043	0.111	0.021	0.116	0.052
Overall	0.106	0.041	0.106	0.021	0.113	0.058
RN						
Left	0.025	0.036	0.074	0.027	0.083	0.014
Right	0.031	0.036	0.074	0.025	0.084	0.020
Overall	0.028	0.036	0.074	0.026	0.083	0.017
STN						
Left	0.026	0.041	0.027	0.047	0.042	0.042
Right	0.029	0.046	0.034	0.025	0.029	0.029
Overall	0.027	0.043	0.030	0.037	0.036	0.036
SN						
Left	0.092	0.031	0.115	0.030	0.124	0.033
Right	0.098	0.036	0.117	0.024	0.141	0.046
Overall	0.095	0.034	0.115	0.026	0.133	0.040
PAG	−0.045	0.030	−0.057	0.017	−0.053	0.015

*STR* striatum, *GPe* globus pallidus externa, *GPi* globus pallidus interna, *RN* red nucleus, *STN* subthalamic nucleus, *SN* substantia nigra, *PAG* periaqueductal grey

### Relationship between qMRI values

**Fig. 5:**
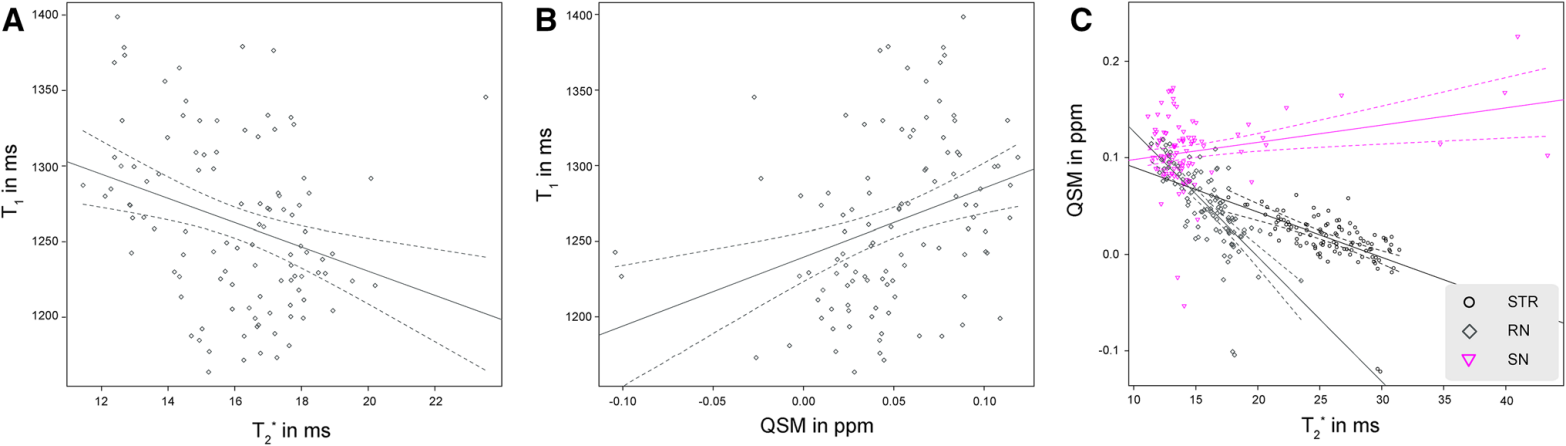
The relationship between qMRI parameters. **a** The $T_{1}$ values versus the $T_{2}^{*}$ values in the striatum. **b** The $T_{1}$ values versus the QSM values in the striatum. **c** The QSM values versus the $T_{2}^{*}$ values in the striatum, red nucleus and substantia nigra. The regression lines indicate a significant correlation between qMRI parameters for a given structure. The dotted lines indicate 95% confidence interval. *STR* striatum, *RN* red nucleus, *RN* substantia nigra, *QSM* quantitative susceptibility mapping

To test whether the $T_{1}$, $T_{2}^{*}$, and QSM values are highly dependent on each other, the different modalities were correlated. For the RN only, the $T_{1}$ values correlated with the $T_{2}^{*}$ (*r* = −0.31, t(104) = −3.30, *p* = 0.009) and QSM values (*r* = 0.32, t(104) = 3.48, *p* = 0.005). For the STR and RN there was a negative correlation between the QSM and $T_{2}^{*}$ values (STR: *r* = −0.57, t(104) = −7.04, *p* < 0.001; RN: *r* = −0.70, t(104) = −10.05, *p* < 0.001), but for the SN, this correlation was positive (*r* = 0.27, t(104) = 2.91, *p* = 0.031). See Fig. 5 for the significant correlations between qMRI values per structure.

### Probability maps

**Fig. 6:**
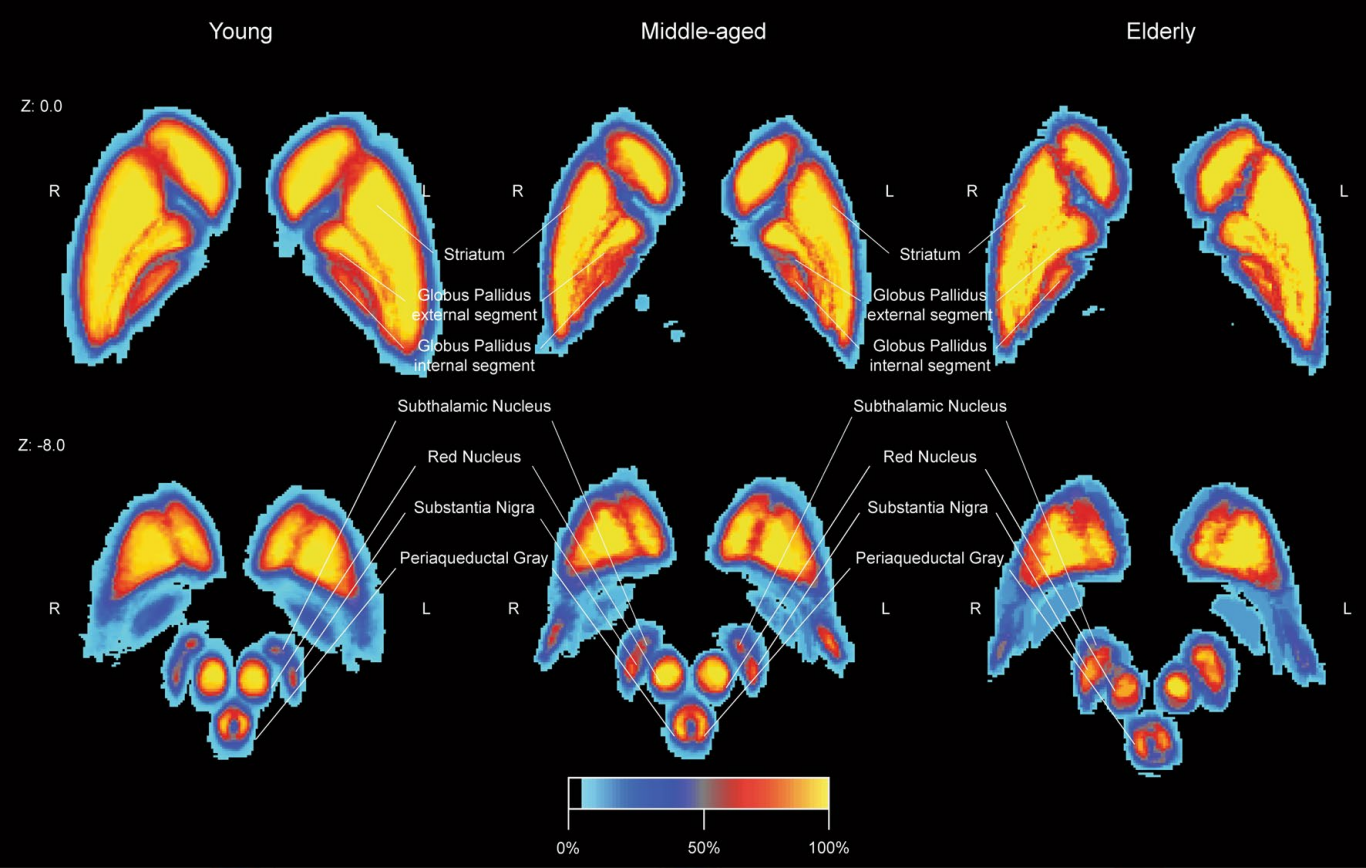
The linearly registered probability atlas in MNI-space per age group. The colour intensity reflects the percentage overlap across the participants per age group. A 100% overlap for the young indicates that 30 participants shared that voxel, whereas a 100% overlap for the elderly indicates that nine participants shared that voxel

Using the linearly registered probability atlases, the maximum and mean percentage overlaps were calculated following (Diedrichsen et al. 2009). Except for the STN, the maximum percentage overlap was generally high across structures and age groups. Similarly, the mean percentage overlap was lowest for the STN compared to the other included structures. This might indicate either larger anatomical or increased registration variability for the STN compared to the other structures. As neighbouring structures of similar size and shape (RN, SN) did not show this trend, anatomical variability seems more plausible. This would need further testing with larger samples and more elaborate shape analysis to fully answer this question. See Table 5 for the maximum and mean overlap per structure and age group. Given that different registration procedures can result in differences in overlap, one would ideally only use the current probability atlases after using similar normalisation protocols (Diedrichsen et al. 2009; Klein et al. 2009). See Fig. 6 for the linearly registered probability atlases per structure and age group.

**Table 5:** The maximum and mean percentage overlap per linearly registered structure and age group

Structure	Young		Middle-aged		Elderly	
	Maximum	Mean	Maximum	Mean	Maximum	Mean
STR						
Left	100.00	42.94	100.00	47.01	100.00	46.82
Right	100.00	42.66	100.00	43.80	100.00	45.47
GPe						
Left	100.00	26.79	100.00	30.88	100.00	35.41
Right	100.00	26.26	100.00	30.10	100.00	35.22
GPi						
Left	100.00	22.09	99.14	26.52	100.00	23.89
Right	99.18	21.82	100.00	25.44	100.00	23.95
RN						
Left	100.00	25.14	100.00	30.87	100.00	28.72
Right	100.00	25.22	100.00	28.37	95.90	28.00
STN						
Left	65.09	9.11	49.27	8.53	61.14	14.24
Right	63.31	9.96	54.38	8.79	53.52	10.81
SN						
Left	90.42	18.10	98.22	22.93	99.40	25.84
Right	78.02	16.70	89.57	20.89	88.89	22.59
PAG	95.31	19.91	99.83	24.48	93.34	21.62

*STR* striatum, *GPe* globus pallidus externa, *GPi* globus pallidus interna, *RN* red nucleus, *STN* subthalamic nucleus, *SN* substantia nigra, *PAG* periaqueductal grey

## Discussion

Using ultra-high field 7T MRI, we show that healthy aging has variable anatomical effects on a number of subcortical structures. In line with previous reports we show that ventricular volume generally increased with age, and that the volume of most subcortical grey matter areas decrease (Barron et al. 1976; Scahill et al. 2003; Terribilli et al. 2011). In addition to volumetric changes the spatial location of several structures was also affected by age. For the STR, GPe, RN, STN, and PAG it was shown that the CoM in MNI space changed with age. This shift in location with age has been previously reported for the STN, using a range of different registration pipelines, but not, to the best of our knowledge, for the STR, GPe, RN, or PAG (Dunnen and Staal 2005; Kitajima et al. 2008; Keuken et al. 2013; Mavridis et al. 2014). These volumetric and positional changes should be taken into account in surgical procedures such as deep brain stimulation (DBS). For instance, one of the frequently targeted DBS structures for Parkinson’s disease is the STN (Deep-Brain Stimulation for Parkinson's Disease Study Group 2001; Follett and Torres-Russotto 2011; Schuepbach et al. 2013). There are several different strategies to determine the location of the stimulation site within the STN, but a prominent procedure is the indirect targeting of the STN using landmarks such as the AC-PC mid commissural point or the RN (Bejjani et al. 2000; Andrade-Souza et al. 2005; Fytagoridis and Blomstedt 2010). Given the changes in volume and location of the RN and STN, one might argue against this procedure of indirect targeting and instead use direct visualization of the STN, ideally with the use of ultra-high field MRI (Cho et al. 2010, 2011; Abosch et al. 2010; Beisteiner et al. 2011). In general, as indicated by the probability atlas, there is a large anatomical variability across the different age groups and structures. These volumetric and positional changes should be taken into account in surgical procedures such as deep brain stimulation (DBS).

In terms of quantitative MRI parameters, we show that for all included subcortical grey matter nuclei, the $T_{1}$ value gradually increased with age, possibly indicating a loss of myelin or incomplete remyelination (Zivadinov 2007; Stüber et al. 2014; Callaghan et al. 2014; Steiger et al. 2016). The $T_{2}^{*}$ and QSM results were more variable and are thought to reflect different aspects of the iron and myelin content in the tissue (Deistung et al. 2013; Stüber et al. 2014). For instance, a decrease in myelin is thought to be indicated with a lengthening of $T_{2}^{*}$ and an increase of QSM values, whereas an increase of iron is considered to shorten $T_{2}^{*}$ and increase quantitative magnetic susceptibility (Deistung et al. 2013). QSM is thought to mainly reflect ferritin-bound iron in grey matter, with an increase in QSM indicating an increased iron concentration (Langkammer et al. 2012; Zheng et al. 2013; Stüber et al. 2014; Ropele and Langkammer 2016).

There was converging evidence for a potential increase of iron concentration with age within the STR and RN, which showed decreased $T_{2}^{*}$ and increased magnetic susceptibility. In addition, there was a negative correlation between the $T_{2}^{*}$ and QSM values, hinting at an increase of iron. This is in line with a recent meta-analysis indicating an aging effect on the accumulation of iron in the STR and RN (Daugherty and Raz 2013). No statistical significant effect of $T_{2}^{*}$ or QSM were found for the GPe, STN, or PAG. The lack of results for the GPe where not surprising as the reported effect size of age on $T_{2}^{*}$ values in the GP are relatively small and might be difficult to find with the current sample size (Daugherty and Raz 2013). The absence of a significant QSM correlation for the GP and no $T_{2}^{*}$ changes in the PAG were reported previously (Lambert et al. 2013; Acosta-Cabronero et al. 2016). It was surprising to find no significant relationship between the $T_{2}^{*}$ values in the STN and age. This contrasts with previous findings by our group using similar segmentation protocols and more recently by whole brain approaches (Keuken et al. 2013; Betts et al. 2016). As all three studies are based on cross-sectional designs with relative small sample sizes, there is a clear need for large sample longitudinal studies to answer the question whether age influences the $T_{2}^{*}$ values in the STN.

The results for the GPi and SN are more challenging to interpret. For the SN, the increased $T_{1}$ indicates a potential decrease in myelin content, and the increased magnetic susceptibility shows a potential increase of iron content. The increase in QSM values in the SN has been reported previously (Bilgic et al. 2012; Gong et al. 2015; Acosta-Cabronero et al. 2016). The decrease of myelin for the SN is further supported by the positive correlation between the $T_{2}^{*}$ and QSM values. For the GPi, an increase of $T_{2}^{*}$ was found but no significant age effects were detected for the QSM values. The increase in $T_{2}^{*}$ is more difficult to explain and is in direct contrast to the meta-analysis of Daugherty and Raz (2013). The increase of $T_{2}^{*}$ may arise from a more uniform distribution of iron within the tissue, or may provide additional evidence for the decrease of myelin (Siemonsen et al. 2008; Deistung et al. 2013).

These changes with age in quantitative MRI values have several methodological implications. The change in $T_{1}$ may well affect the accuracy of a number of automatic parcellation tools, such as FIRST or Freesurfer, which have been used to investigate volumetric changes in the subcortex with aging (e.g., Goodro et al. 2012; Liem et al. 2015). These parcellation tools are solely based on the contrast of a $T_{1}$-weighted image (Visser et al. 2016a) and could thus easily be influence by changes in the value of $T_{1}$ (Jernigan et al. 2001). Previous work by Wenger et al. (2014) and Lorio et al. (2014, 2016b) have indeed shown an age-related bias in automatic segmentation and that certain sequences are less prone to this age effect (Helms et al. 2009).

The change of $T_{2}^{*}$ values might affect the sensitivity in fMRI studies. Since the optimal TE in a gradient-echo EPI sequence is equal to the $T_{2}^{*}$ relaxation time, an effect of aging on the $T_{2}^{*}$ values results in a change of BOLD sensitivity (Ugurbil et al. 2007; Koopmans et al. 2011; Norris 2012). It might be the case that when fMRI data is acquired for two age groups with the same TE this might result in a significant apparent difference which might actually be driven by changes in anatomy but not necessarily in function (e.g., Mell 2009; Eppinger et al. 2013).

## Limitations

As with any cross-sectional study there is always the risk for cohort effects. Particularly when a small sample is used, it is not trivial to attribute the observed effects to individual variability or to healthy aging. The number of healthy elderly subjects that underwent all the UHF-MRI scans was relatively low and might be the cause of relatively low correlation coefficients. While this is true, it should be noted that cross-sectional and longitudinal studies generally show similar effects of aging, while the effects are more pronounced in longitudinal studies (Scahill et al. 2003). Another limitation is that the structures were parcellated using only one MRI contrast, which for most structures was shown to change with age. Though unlikely, the changes in $T_{1}$, $T_{2}^{*}$, and QSM values could have resulted in a shift of the perceptual boundaries, influencing the volumetric results, while the underlying true anatomy remained stable. A potential solution for this is to use multiple contrasts simultaneously to inform the parcellation. Recent parcellation tools have indeed shown that the combination of multiple MRI contrasts in a Bayesian framework improve the segmentation of subcortical structures (Kim et al. 2014; Visser et al. 2016b, a; Lorio et al. 2016a).

The estimation of the qMRI values and the relationship with the underlying tissue composition is not trivial (Weiskopf et al. 2015; Ropele and Langkammer 2016). In the current study the $T_{1}$ values were estimated using a well-characterized and tested MP2RAGE sequence, but it is acknowledged that the resulting $T_{1}$ maps may contain a residual transmit field bias which would result in less precise measures (Lutti et al. 2014). Nonetheless, the reported $T_{1}$ values of the GP are similar to a recent 7T multi-site test–retest validation study indicating that the $T_{1}$ values estimated from the MP2RAGE are stable (Voelker et al. 2016). The $T_{2}^{*}$ values were estimated by fitting a mono-exponential fit on a multi-echo $T_{2}^{*}$-weighted volume (Chavhan et al. 2009) but it is known that the $T_{2}^{*}$ values are influenced by the choice of TE, number of TE’s, and the actual function used to estimate the $T_{2}^{*}$ values (Yin et al. 2010; Milford et al. 2015). Although different methods were used to estimate $T_{2}^{*}$, the current $T_{2}^{*}$ values for the STR, GP, SN, and RN resulted in a similar rank ordering as previous 7T MRI work (Khabipova et al. 2015). The same holds for the calculation of QSM values. These values are influenced by a number of variables during both the acquisition phase and the post-processing phase (Haacke et al. 2015; Ropele and Langkammer 2016).

While ultra-high field MRI has several benefits for imaging small subcortical structures it is not without methodological challenges (van der Zwaag et al. 2015). As the field strength increases, the B_0_ inhomogeneity increases as well resulting in an inhomogeneous tissue contrast (van de Moortele et al. 2005; van der Zwaag et al. 2015). However, the autoshim facility of the 7T MRI scanner used is normally observed to provide a satisfactory shim, and is comparable to other 7T MRI sites (Voelker et al. 2016).

A final limitation is the low number of parcellated subcortical structures, given the total number of structures that are known to exist (Alkemade et al. 2013). We included only a small number of structures for several limiting reasons. The first one is the required time for careful manual delineation of each individual structure. The second, but more important, reason is that we selected only those structures that were easily visible with the contrast and spatial resolution employed. To discriminate more structures such as the thalamic nuclei, it may help to use specific MRI sequences that are tailored to that structure (Tourdias et al. 2014), improve the spatial resolution, or consider *post mortem* studies (Keren et al. 2015; Forstmann et al. in press). By reducing the voxel size, the partial volume effects (PVE) become less prominent. As the voxel resolution improves, the correction for subject motion becomes increasingly important and needs to be taken into account during the data acquisition (Tisdall et al. 2016). Prospective motion correction would additionally reduce the PVE and result in a higher SNR while allowing for higher spatial resolution (e.g., Stucht et al. 2015; Federau and Gallichan 2016). The need for reducing the PVE is crucial for smaller structures as their surface-area-to-volume ratio is larger (Vos et al. 2011).

## Conclusion

Using quantitative ultra-high field 7T MRI, we were able to show variable age-related changes in anatomical features in several subcortical nuclei, as well as age-related changes in underlying MRI parameters that drive typical image contrast. These qMRI changes seem to be driven by different mechanisms: the increase of $T_{1}$ values indicate a global decrease of myelination across the subcortical structures, whereas some of the structures, $T_{2}^{*}$ and QSM results indicate an iron accumulation with age.
